# Concomitant exposure to benzodiazepines during pembrolizumab-based therapy for advanced non-small-cell lung cancer: a propensity-score matched analysis of monitoring agency data

**DOI:** 10.37349/etat.2025.1002287

**Published:** 2025-01-20

**Authors:** Fabrizio Nelli, Enzo Maria Ruggeri, Antonella Virtuoso, Diana Giannarelli, Armando Raso, Federica Natoni, Gloria Pessina, Daniele Remotti, Mario Giovanni Chilelli, Carlo Signorelli, Agnese Fabbri

**Affiliations:** University of Salford, UK; ^1^Department of Oncology and Hematology, Thoracic Oncology Unit, Central Hospital of Belcolle, 01100 Viterbo, Italy; ^2^Department of Oncology and Hematology, Medical Oncology Unit, Central Hospital of Belcolle, 01100 Viterbo, Italy; ^3^Biostatistics Unit, Scientific Directorate, Fondazione Policlinico Universitario A. Gemelli, IRCCS, 00136 Rome, Italy; ^4^Department of Oncology and Hematology, Thoracic and Interventional Radiology, Central Hospital of Belcolle, 01100 Viterbo, Italy; ^5^Department of Oncology and Hematology, Molecular Biology and Genetics, Central Hospital of Belcolle, 0100 Viterbo, Italy; ^6^Department of Oncology and Hematology, Pathology Unit, Central Hospital of Belcolle, 01100 Viterbo, Italy

**Keywords:** Concomitant medications, non-small-cell lung cancer, immune checkpoint blockade, pembrolizumab, chemotherapy, first-line therapy, benzodiazepines, efficacy

## Abstract

**Aim:**

The interaction of concomitant benzodiazepine (BZD) exposure during immune checkpoint blockade has not been comprehensively investigated to date. This research aimed to determine the influence of BZD intake on the survival outcomes of patients with metastatic non-small-cell lung cancer (NSCLC) receiving pembrolizumab-based therapies.

**Methods:**

We included consecutive patients with advanced NSCLC who were given frontline pembrolizumab, whether as exclusive therapy or combined with platinum-based chemotherapy. The classification of BZD relied on the molecular composition, distinguishing between *N*-substituted and *N*-unsubstituted compounds.

**Results:**

During the time frame from April 2018 to May 2023, we enrolled 258 patients, 156 (60.5%) and 102 (39.5%) of whom received pembrolizumab alone or the combination regimen, respectively. We identified 108 (41.8%) exposed patients (BZD cohort) in comparison to all others (no-BZD cohort). After applying propensity-score matching, 108 cases were relevant for each cohort. After a median follow-up of 16.3 [95% confidence interval (CI) 13.1–19.7] months, univariate analysis revealed no significant differences in terms of progression-free survival (PFS) or overall survival (OS) between BZD cohorts. However, patients exposed to *N*-substituted compounds had significantly longer PFS and OS than those who did not take BZD. Conversely, patients exposed to *N*-unsubstituted compounds experienced significantly shortened OS. Multivariate testing showed that taking unspecified BZD had no impact on PFS or OS, while *N*-substituted BZD exposure correlated independently with longer PFS [hazard ratio (HR) 0.52 (95% CI 0.34–0.79); *P* = 0.002] and OS [HR 0.58 (95% CI 0.38–0.88); *P* < 0.001]. In contrast, *N*-unsubstituted BZD intake had worsening effects on OS [HR 1.92 (95% CI 1.20–3.06); *P* = 0.006].

**Conclusions:**

BZD exposure may impact the efficacy of immune checkpoint inhibitors in patients with advanced NSCLC. The specific composition may influence the choice among different compounds.

## Introduction

Cancer immunology has recently made significant progress in understanding the role of γ-aminobutyric acid (GABA) signaling [1]. Although GABA is mainly recognized as an inhibitory neurotransmitter, emerging research indicates that it also has a crucial role in controlling the growth and metastasis of tumor cells and the immune response within the tumor microenvironment (TME) [2]. GABA acts as a ligand that modulates the activity of various types of receptors, including ionotropic A (GABA_A_) receptors and metabotropic B (GABA_B_) receptors [3]. Previous research has demonstrated that immune cells express GABA receptors and their activation through GABA binding can suppress the immune response by inhibiting CD4^+^ T helper and CD8^+^ T cytotoxic cells, increasing regulatory T cells, and reducing the pro-inflammatory properties of antigen-presenting cells [4]. The relevance of GABA in modulating the TME has been highlighted by two landmark preclinical studies. These findings suggest that GABAergic signaling can directly impact the TME, potentially preventing the recruitment of dendritic cells and promoting tumor progression in an autocrine manner, while also inhibiting the infiltration of antitumor immune cells in a paracrine manner. Genetic or pharmacological inhibition of GABA production could synergize with immune checkpoint blockade to overcome tumor resistance to immunotherapy [5, 6].

Benzodiazepines (BZDs), barbiturates, neurosteroids, and certain anesthetics are commonly used pharmacological agents that target GABA receptors in clinical practice. BZDs, in particular, are psychoactive drugs with various properties, such as sedation, hypnosis, anxiety relief, anticonvulsant effects, and muscle relaxation [7]. These class effects and specific therapeutic indications, including the control of anticipatory nausea [8] and dyspnea [9], make BZDs the most frequently prescribed psychoactive medications for advanced cancer patients. The effects of BZDs depend on the allosteric modulation of central and peripheral binding sites. While the central receptor is part of the GABA_A_ receptor complex [10], the peripheral receptor, known as translocator protein 18 kDa (TSPO), is a protein located in the external mitochondrial membrane. TSPO is expressed in a wide range of cell types, including platelets and several immune cells. Signaling mediated by TSPO is involved in processes such as apoptosis, cell proliferation, differentiation, mitochondrial function regulation, and immunomodulation [11]. As regards the latter, recent preclinical studies have renewed interest in the effects of BZDs on different phases of the immune response. Positive allosteric modulation of GABA_A_ receptors and stimulation of TSPO from BZDs dampers the activation of innate cells and hinders the onset and development of adaptive inflammatory responses [12, 13]. From a clinical perspective, several population-based studies have reported increased susceptibility to spontaneous microbial infections and mortality in relation to BZD use [14, 15].

There is a growing recognition that the efficacy of immune checkpoint blockade can be influenced, either positively or negatively, by commonly prescribed medications [16]. Ongoing research is exploring these interactions, providing insights to improve patient outcomes [17]. Despite the available evidence suggesting that BZD treatment has immunosuppressive properties, so far no study has investigated the potential interaction between these drugs and immune checkpoint inhibitors. It would be worthwhile to thoroughly examine the potential interaction between BZDs and immune checkpoint inhibitors. We therefore sought to investigate the association between concomitant BZD exposure and clinical outcomes in patients with advanced non-small-cell lung cancer (NSCLC) undergoing first-line immune checkpoint blockade with or without chemotherapy. The current research also aimed to determine whether taking specific BZDs could affect survival in the same patient population. In this regard, we adopted a classification of BZDs based on their molecular structure, which is hypothesized to exert different modulatory effects on the immune system, distinguishing between *N*-substituted and *N*-unsubstituted compounds [18].

## Materials and methods

### Study design and eligibility

The study retrospectively involved patients with metastatic NSCLC participating in the national registry for prospective monitoring of high-cost drug accountability [19]. The key eligibility criteria were histologically proven diagnosis of NSCLC, Eastern Cooperative Oncology Group Performance Status (ECOG PS) of 0–2, and receipt of pembrolizumab alone or in combination with platinum-based chemotherapy as frontline treatment lasting a minimum of two cycles. The study allowed patients with asymptomatic or neurologically stable brain metastases to participate. Patients who had received perioperative chemotherapy after previous thoracic surgery and/or radiotherapy were included if disease recurrence occurred more than six months after the end of treatment. Conversely, exclusion criteria were unknown programmed cell death ligand-1 (PD-L1) tumor proportion score (TPS), actionable mutations involving the *EGFR*, *BRAF*, *ALK*, or *ROS-1* genes, recent exposure to high-dose corticosteroids or other immunosuppressants, or previous treatment with anti-PD-(L)1 inhibitors. The research project was reviewed and approved by the relevant Ethics Committee (registration code: Oss-R-281; protocol number: 855/CE Lazio1) and adhered to the STROBE guidelines for observational studies. All participants gave written informed consent, which allowed their de-identified personal information to be used for clinical research.

### Data source and assessments

The National Drug Agency was the source of demographic data, clinical, pathological, and molecular characteristics of patients, as well as treatment outcomes concerning disease response and survival rates [19]. Immunohistochemical assessment of PD-L1 TPS relied on the standard procedure involving the anti-PD-L1 22C3 pharmDx antibody (Agilent Technologies, Inc., Santa Clara, CA) [20]. We referred to internal health records for laboratory tests to calculate the lung prognostic immune index (LIPI) [21] and information on concomitant medications. BZD exposure was defined as a continuous intake of at least 30 days in the time frame ranging from 30 days before to 60 days after the initial administration of pembrolizumab. We also noted the specific types of BZDs (*N*-unsubstituted vs. *N*-substituted) and their therapeutic indication [22]. We also examined the use of other drugs that could potentially affect the efficacy of immune checkpoint blockade, including corticosteroids, systemic antibiotics, and the intake of acetaminophen (APAP) and proton pump inhibitors (PPI). Our primary endpoint was to evaluate the impact of generic BZD exposure on progression-free survival (PFS) and overall survival (OS). Additionally, the study aimed to assess the effects of specific types of BZDs on these outcomes. Baseline disease assessments were performed within 4 weeks of treatment initiation, followed by regular re-evaluations every 12 weeks [19]. A blinded radiologist reviewed patient records using the iRECIST criteria [23]. PFS was calculated from the start of pembrolizumab treatment until documented disease progression or death without evidence of disease progression. OS was calculated from the start of pembrolizumab treatment until death, regardless of the cause. Patients whose disease did not progress and who were still surviving by the last follow-up were censored as of May 31, 2024.

### Statistical analysis

The current research applied SPSS Statistics for Windows (Version 23.0. Armonk, NY: IBM Corp.) and GraphPad Prism version 9.0 (GraphPad Software, Boston, MA) for all statistical evaluations and figure rendering, respectively. The sample size calculator for matched case-control observational studies was applied to estimate the number of patients to be included in the current analysis [24]. The selected design parameters *P0* (the ratio of patients with expected disease progression in the non-exposed cohort) and *P1* (the ratio of patients with expected disease progression in the exposed cohort) were set at 0.35 and 0.52, respectively. Given an alpha and beta error probability of 0.05 and 0.80, respectively, and a non-exposed/exposed ratio of 1.0, both cohorts required at least 106 cases. Descriptive analyses included calculating the mean with standard deviation (SD) for categorical variables or frequencies (absolute and relative) with interquartile range (IQR) or 95% CI for continuous variables. The description of patient characteristics at baseline relied on generic BZD exposure. We used propensity score matching (PSM) to balance the baseline characteristics between the study cohorts. We estimated the propensity scores through a multivariate logistic regression model that included all potentially prognostic factors [age, sex, ECOG PS, histology, disease extent, metastatic sites, PD-L1 TPS, body mass index (BMI), smoking habits, previous radiation therapy, LIPI score, treatment regimen, and relevant concomitant medications]. We applied a 1:1 matching algorithm with a caliper width of 0.2 to ensure equal representation in both subgroups. We evaluated the balance of baseline risk factors before and after PSM using appropriate comparison tests, such as Pearson’s *χ*^2^, Mann-Whitney *U*, or the Kruskal-Wallis tests. The balance of covariates between study cohorts was further assessed by calculating the standardized mean difference (SMD), with a value less than 0.1 indicating a well-balanced outcome [25]. The PSM process utilized R software version 4.1.2 and the MatchIt library [26]. PFS and OS were estimated and compared using the Kaplan-Meier method and a two-sided log-rank test, respectively. A multivariate Cox regression model was applied to calculate hazard ratio (HR) with 95% CI and compare the incidences of disease progression and death. To mitigate alpha inflation risk from univariate multiple comparisons, multivariate analysis included generic and specific BZD exposure categories in addition to all variables used to estimate propensity scores. All tests were two-tailed, and a *P*-value less than 0.05 was considered statistically significant.

## Results

### Patient characteristics

A total of 258 patients met the inclusion criteria for the current analysis. Between April 2018 and May 2023, 156 (60.5%) received pembrolizumab alone. From November 2019 to May 2023, 102 (39.5%) patients were given pembrolizumab combined with platinum-based chemotherapy. We identified 108 (42.2%) patients taking BZDs, which represented the exposed group (BZD-cohort) compared to all others (no-BZD cohort). Univariate comparison of baseline characteristics showed a significant imbalance in the distribution of pharmacological variables, concerning significantly higher APAP and PPI intake for patients in the no-BZD cohort. Applying a comprehensive PSM, we obtained a homogeneous distribution of covariates across subgroups. The final analysis involved 108 patients from each cohort. Table 1 depicts the characteristics of both populations at baseline according to BZD exposure.

**Table 1:** Patient characteristics

**Variable**	**Unadjusted population**					**PSM-adjusted population**				
	**All patients (*N* = 258)**	**No-BZD cohort (*N* = 150)**	**BZD cohort (*N* = 108)**	***P* value**	**SMD**	**All patients (*N* = 216)**	**No-BZD cohort** **(*N* = 108)**	**BZD cohort (*N* = 108)**	***P* value**	**SMD**
Age										
- Mean (SD), years	70.0 (8.4)	70.0 (7.9)	69.5 (9.0)	0.332	-	70.0 (8.43)	70.0 (7.77)	69.5 (9.04)	0.457	-
- ≥ 70 years	136 (52.7%)	82 (54.7%)	54 (50.0%)	0.459	0.046	111 (51.4%)	57 (52.8%)	54 (50.0%)	0.685	0.027
Sex										
- Female	78 (30.2%)	40 (26.7%)	38 (35.2%)	0.207	0.093	74 (34.3%)	36 (33.3%)	38 (35.2%)	0.776	0.018
- Male	180 (69.8%)	110 (73.3%)	70 (64.8%)			142 (65.7%)	72 (66.7%)	70 (64.8%)		
ECOG PS										
- 0 or 1	208 (80.6%)	120 (80.0%)	88 (81.5%)	0.766	0.014	175 (81.0%)	87 (80.6%)	88 (81.5%)	0.863	0.009
- 2	50 (19.4%)	30 (20.0%)	20 (18.5%)			41 (19.0%)	21 (19.4%)	20 (18.5%)		
Histologic subtype										
- Nonsquamous	202 (78.3%)	118 (78.7%)	84 (77.8%)	0.864	< 0.001	167 (77.3%)	83 (76.9%)	84 (77.8%)	0.872	0.009
- Squamous	56 (21.7%)	32 (21.3%)	24 (22.2%)			49 (22.7%)	25 (23.1%)	24 (22.2%)		
No. of metastatic sites										
- ≤ 2	138 (53.5%)	79 (52.7%)	59 (54.6%)	0.755	0.019	120 (55.6%)	61 (56.5%)	59 (54.6%)	0.785	0.067
- > 2	120 (46.5%)	71 (47.3%)	49 (45.4%)			96 (44.4%)	47 (43.5%)	49 (45.4%)		
Bone metastasis	53 (20.5%)	30 (19.1%)	23 (21.3%)	0.799	< 0.001	44 (20.4%)	21 (19.4%)	23 (21.3%)	0.737	0.055
Brain metastasis	58 (22.5%)	30 (20.0%)	28 (25.9%)	0.261	< 0.001	55 (25.5%)	27 (25.0%)	28 (25.9%)	0.877	0.059
Liver metastasis	28 (10.9%)	19 (12.7%)	9 (8.3%)	0.270	0.043	20 (9.3%)	11 (10.2%)	9 (8.3%)	0.641	0.039
PD-L1 TPS										
- < 1%	80 (31.0%)	45 (30.0%)	35 (32.4%)	0.066	0.326	71 (32.9%)	36 (33.3%)	35 (32.4%)	0.663	0.083
- ≥ 1% and ≤ 49%	22 (8.5%)	8 (5.3%)	14 (13.0%)			22 (10.2%)	8 (7.4%)	14 (13.0%)		
- ≥ 50%	156 (60.5%)	97 (64.7%)	59 (54.6%)			123 (56.9%)	64 (59.3%)	59 (54.6%)		
BMI										
- Mean (SD), kg/m^2^	25.1 (4.80)	24.8 (5.0)	25.6 (4.5)	0.635	< 0.001	25.9 (4.87)	26.0 (5.15)	25.6 (4.55)	0.233	-
- ≥ 25	130 (50.4%)	73 (48.7%)	57 (52.8%)	0.515		114 (52.8%)	57 (52.8%)	57 (52.8%)	0.999	< 0.001
Smoking habits										
- Never	23 (8.9%)	14 (9.3%)	9 (8.3%)	0.781	< 0.001	20 (9.3%)	11 (10.2%)	9 (8.3%)	0.641	< 0.001
- Ever	235 (91.1%)	136 (90.7%)	99 (91.7%)			196 (90.7%)	97 (89.8%)	99 (91.7%)		
Previous thoracic RT	35 (13.6%)	16 (10.7%)	19 (17.6%)	0.109	< 0.001	28 (13.0%)	9 (8.3%)	19 (17.6%)	0.043	< 0.001
LIPI score										
- 0	100 (38.8%)	60 (40.0%)	40 (37.0%)	0.740	< 0.001	84 (38.9%)	44 (40.7%)	40 (37.0%)	0.742	< 0.001
- 1	88 (34.1%)	52 (34.7%)	36 (33.3%)			68 (31.5%)	32 (29.6%)	36 (33.3%)		
- 2	70 (27.1%)	38 (25.3%)	32 (29.7%)			64 (29.6%)	32 (29.6%)	32 (29.6%)		
First-line therapy										
- Only pembrolizumab	156 (60.5%)	96 (64.0%)	60 (55.6%)	0.377	< 0.001	124 (57.4%)	64 (59.3%)	60 (55.6%)	0.596	< 0.001
- Pemetrexed-based	84 (32.6%)	45 (30.0%)	39 (36.1%)			75 (34.7%)	36 (33.3%)	39 (36.1%)		
- Paclitaxel-based	18 (7.0%)	9 (6.0%)	9 (8.3%)			17 (7.9%)	8 (7.4%)	9 (8.3%)		
Corticosteroids^a^	103 (39.9%)	54 (36.0%)	49 (45.4%)	0.129	< 0.001	94 (43.5%)	45 (41.7%)	49 (45.4%)	0.585	< 0.001
APAP^b^	101 (39.1%)	67 (44.7%)	34 (31.5%)	0.032	0.131	62 (28.7%)	28 (25.9%)	34 (31.5%)	0.369	< 0.001
Systemic antibiotics^c^	58 (22.5%)	38 (25.3%)	20 (18.5%)	0.196	0.068	40 (18.5%)	20 (18.5%)	20 (18.5%)	0.999	< 0.001
PPI^d^	88 (34.1%)	59 (39.3%)	29 (26.9%)	0.036	0.124	58 (26.9%)	29 (26.9%)	29 (26.9%)	0.999	< 0.001
Specific BZD intake										
- *N*-substituted^e^	57 (22.1%)	-	57 (52.8%)	-	-	57 (26.4%)	-	57 (52.8%)	-	-
- *N*-unsubstituted^f^	51 (19.7%)		51 (47.2%)			51 (23.6%)		51 (47.2%)		

PSM: propensity score matching; BZD: benzodiazepine; SMD: standardized mean difference; SD: standard deviation; ECOG PS: Eastern Cooperative Oncology Group Performance Status; PD-L1 TPS: programmed cell death ligand-1 tumor proportion score; BMI: body mass index; RT: radiotherapy; LIPI: lung immune prognostic index; APAP: acetaminophen; PPI: proton pump inhibitors. ^a^ Corticosteroids refer to the use of prednisone or an equivalent drug at a dose of at least 10 mg per day for at least 5 days within the 30 days before the start of treatment, excluding premedication for chemotherapy; ^b^ APAP refers to the use of at least 1,000 mg per day for more than 24 h during the 30 days before the start of treatment; ^c^ systemic antibiotics and ^d^ PPI refer to the use of these medications in the 30 days before the start of treatment; ^e^ *N*-substituted denoted intake of alprazolam, diazepam, bromazepam, or triazolam; ^f^ *N*-unsubstituted denoted intake of lorazepam, clonazepam, delorazepam, or lormetazepam

### Analysis of BZD exposure

Among the 108 exposed patients, 57 (52.8%) were being treated with *N*-substituted BZDs, such as alprazolam, diazepam, bromazepam, or triazolam, while 51 (47.2%) were taking *N*-unsubstituted BZDs, including lorazepam, clonazepam, delorazepam, or lormetazepam. The therapeutic indications, median duration of therapy before the onset of immune checkpoint blockade, and most clinical and pathological features did not differ significantly between the two subgroups. However, previous chest radiotherapy and a low disease burden were significantly more frequent among patients treated with *N*-substituted BZDs (Table S1). We conducted a thorough examination of the factors related to patients’ intake of specific BZDs using logistic regression analysis. Our multivariate models found that patients with a history of *N*-unsubstituted BZD exposure were significantly more likely to be younger than 70 years old, have more than two metastatic sites, not have brain metastases, and not have undergone previous chest radiotherapy (Table S2).

### Treatment outcomes

**Figure 1:**
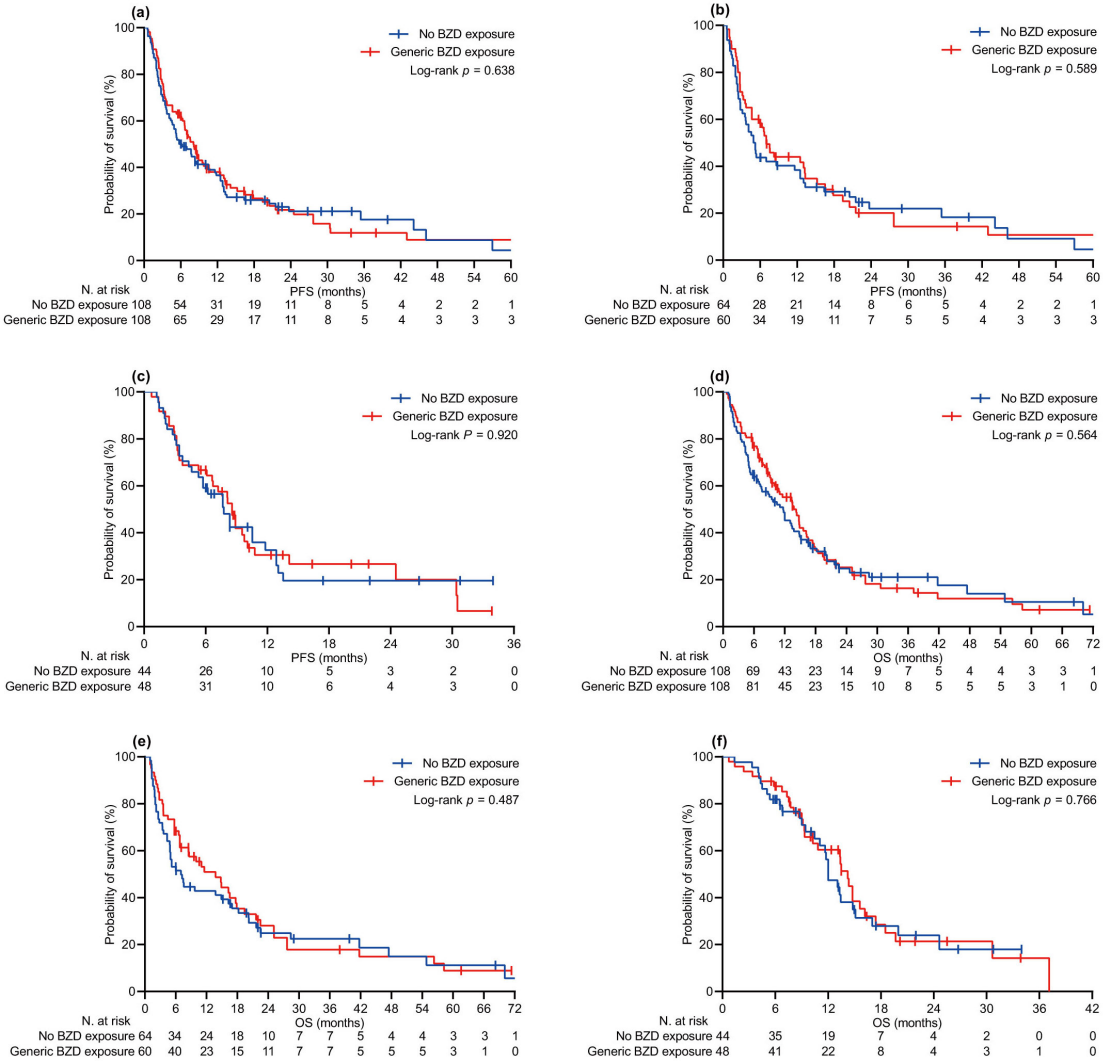
**Kaplan-Meier curves of survival by generic BZD exposure**. (**a**) PFS in the PSM-adjusted population, all patients; (**b**) PFS in patients with PD-L1 TPS ≥ 50% receiving pembrolizumab alone; (**c**) PFS in patients with PD-L1 TPS < 50% receiving pembrolizumab and platinum-based chemotherapy; (**d**) OS in the PSM-adjusted population, all patients; (**e**) OS in patients with PD-L1 TPS ≥ 50% receiving pembrolizumab alone; (**f**) OS in patients with PD-L1 TPS < 50% receiving pembrolizumab and platinum-based chemotherapy. PFS: progression-free survival; OS: overall survival; BZD: benzodiazepine; PD-L1 TPS: programmed cell death ligand-1 tumor proportion score; PSM: propensity score matching

**Figure 2:**
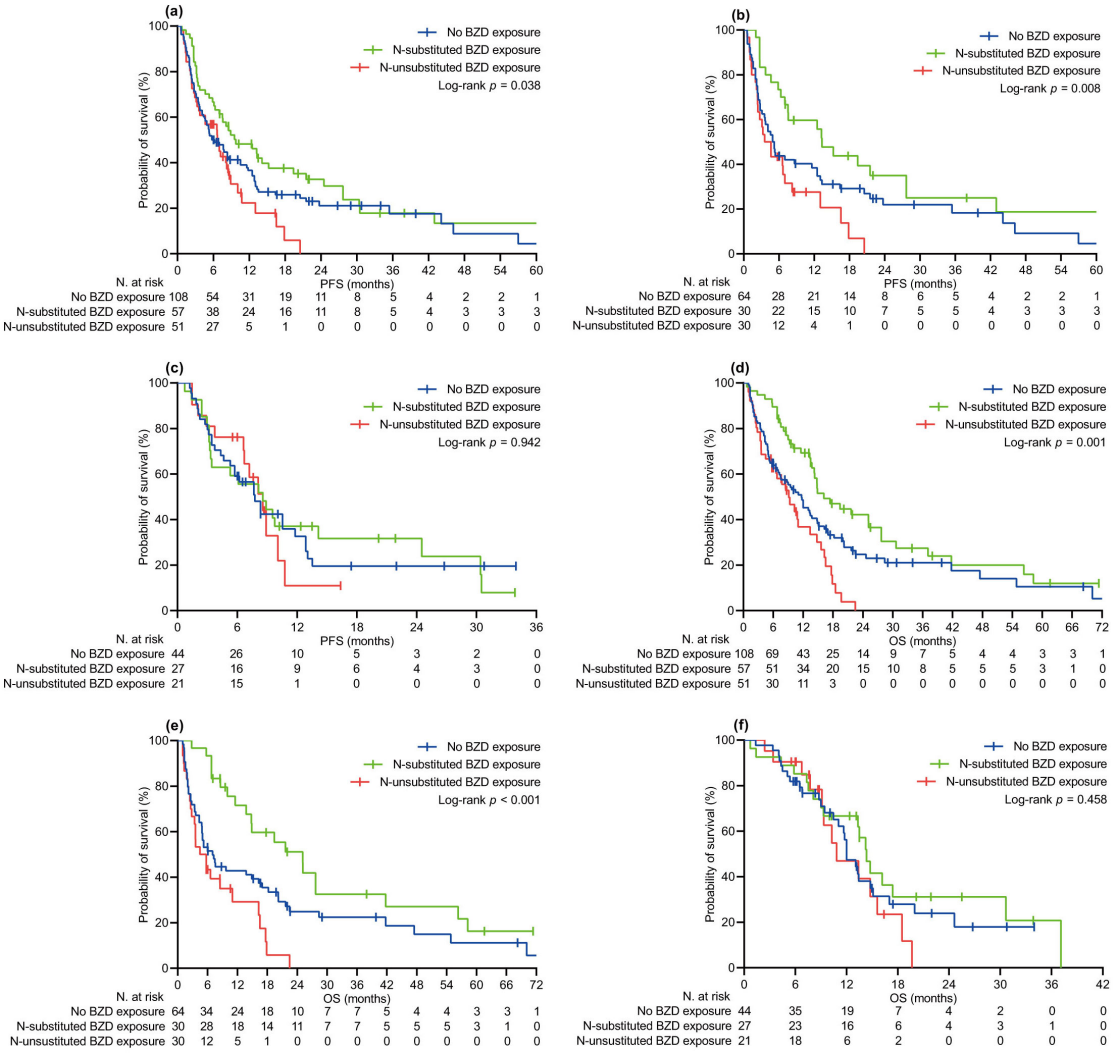
**Kaplan-Meier curves of survival by specific BZD exposure**. (**a**) PFS in the PSM-adjusted population, all patients; (**b**) PFS in patients with PD-L1 TPS ≥ 50% receiving pembrolizumab alone; (**c**) PFS in patients with PD-L1 TPS < 50% receiving pembrolizumab and platinum-based chemotherapy; (**d**) OS in the PSM-adjusted population, all patients; (**e**) OS in patients with PD-L1 TPS ≥ 50% receiving pembrolizumab alone; (**f**) OS in patients with PD-L1 TPS < 50% receiving pembrolizumab and platinum-based chemotherapy. PFS: progression-free survival; OS: overall survival; BZD: benzodiazepine; PD-L1 TPS: programmed cell death ligand-1 tumor proportion score; PSM: propensity score matching. *N*-substituted BZD exposure indicates intake of alprazolam, diazepam, bromazepam, or triazolam. *N*-unsubstituted BZD exposure indicates intake of lorazepam, clonazepam, delorazepam, or lormetazepam

The median follow-up time was 16.3 (95% CI 13.1–19.7) months in the relevant population, with no difference between the BZD [15.0 (95% CI 11.5–22.2) months] and no-BZD cohorts [16.5 (95% CI 12.2–20.4) months, *P* = 0.380]. At the specified time point, we censored 54 (21.1%) and 62 (24.2%) patients who had no evidence of disease progression or were still surviving, respectively. Univariate comparison of PFS and OS involved generic (Figure 1) and specific exposure to BZDs (Figure 2). Generic BZD intake did not affect PFS (Table 2 and Figure 1A) or OS (Table 3 and Figure 1D). Evaluation across treatment subgroups (pembrolizumab alone or combined with cytotoxic chemotherapy) confirmed the lack of a significant difference between the two cohorts for both PFS (Figure 1B and C) and OS (Figure 1E and F). However, when BZDs were categorized according to their molecular composition, patients who were exposed to *N*-substituted compounds experienced significantly longer PFS (Table 2 and Figure 2A) and OS (Table 3 and Figure 2D) than those without a history of BZD intake. In the same comparative assessment, patients exposed to *N*-unsubstituted BZDs had significantly shortened OS (Table 3 and Figure 2D). Further univariate analysis confirmed that patients treated with *N*-substituted BZDs had a significant advantage in terms of PFS and OS, specifically in the subgroup of patients with a high PD-L1 TPS who received pembrolizumab alone (Figures 2B and E). We observed no difference in survival outcomes for the subgroup of patients with PD-L1 TPS ≤ 50% who were treated with pembrolizumab and chemotherapy (Figure 2C and F). The multivariate regression model confirmed that unspecified BZD intake had no independent effect on PFS or OS. According to the same testing, a history of exposure to *N*-substituted BZDs correlated independently with longer PFS and OS. In contrast, taking *N*-unsubstituted BZDs was confirmed to correlate with worse OS (Tables 2 and 3).

**Table 2:** Analysis of PFS

**Covariate**	**Median PFS, months (95% CI)**	**Univariate analysis**		**Multivariate analysis**	
		**HR (95% CI)**	***P* value**	**HR (95% CI)**	***P* value**
Age					
- < 70 years (*N* = 105)	7.0 (4.3–9.6)	1.00	-	1.00	-
- ≥ 70 years (*N* = 111)	7.6 (6.2–9.1)	0.83 (0.61–1.13)	0.240	0.81 (0.57–1.16)	0.260
Sex					
- Female (*N* = 74)	7.7 (4.2–11.3)	1.00	-	1.00	-
- Male (*N* = 142)	7.2 (5.6–8.8)	0.78 (0.54–1.07)	0.130	0.78 (0.54–1.13)	0.200
ECOG PS					
- 0–1 (*N* = 175)	8.1 (6.7–9.5)	1.00	-	1.00	-
- 2 (*N* = 41)	3.3 (2.5–4.1)	1.29 (0.86–1.92)	0.210	1.08 (0.68–1.72)	0.734
Histologic subtype					
- Nonsquamous (*N* = 167)	6.7 (4.4–8.9)	1.00	-	1.00	-
- Squamous (*N* = 49)	13.2 (4.9–21.5)	0.60 (0.40–0.90)	0.015	0.45 (0.27–0.75)	0.003
No. of metastatic sites					
- ≤ 2 (*N* = 120)	8.6 (5.4–11.7)	1.00	-	1.00	-
- > 2 (*N* = 96)	4.3 (1.2–7.4)	1.50 (1.10–2.05)	0.010	2.04 (1.15–3.61)	0.014
Bone metastasis					
- No (*N* = 172)	8.1 (6.7–9.5)	1.00	-	1.00	-
- Yes (*N* = 44)	3.4 (1.9–5.0)	1.68 (1.16–2.43)	0.006	0.94 (0.57–1.55)	0.833
Brain metastasis					
- No (*N* = 161)	7.5 (5.8–9.3)	1.00	-	1.00	-
- Yes (*N* = 55)	7.0 (2.5–11.5)	1.01 (0.71–1.44)	0.943	0.50 (0.29–0.86)	0.013
Liver metastasis					
- No (*N* = 196)	7.7 (6.3–9.0)	1.00	-	1.00	-
- Yes (*N* = 20)	3.4 (2.3–4.5)	1.39 (0.83–2.30)	0.201	1.08 (0.58–2.00)	0.794
PD-L1 TPS					
- < 1% (*N* = 71)	8.1 (7.0–9.2)	1.00	-	1.00	-
- ≥ 1% and ≤ 49% (*N* = 22)	8.5 (2.9–14.2)	1.07 (0.61–1.86)	0.799	1.95 (1.03–3.69)	0.039
- ≥ 50% (*N* = 123)	6.6 (4.5–8.7)	1.04 (0.73–1.48)	0.806	2.99 (0.35–25.32)	0.315
BMI					
- < 25 kg/m^2^ (*N* = 102)	5.4 (2.4–8.3)	1.00	-	1.00	-
- ≥ 25 kg/m^2^ (*N* = 114)	9.7 (6.6–12.9)	0.61 (0.45–0.84)	0.003	0.75 (0.52–1.08)	0.130
Smoking habits					
- Never (*N* = 20)	3.4 (2.0–4.8)	1.00	-	1.00	-
- Ever (*N* = 196)	7.7 (6.3–9.2)	0.44 (0.27–0.72)	0.001	0.93 (0.52–1.67)	0.818
Previous thoracic RT					
- No (*N* = 188)	7.2 (5.2–9.2)	1.00	-	1.00	-
- Yes (*N* = 28)	7.7 (1.5–14.8)	0.78 (0.50–1.23)	0.294	0.63 (0.38–1.04)	0.073
LIPI score					
- 0 (*N* = 84)	24.5 (17.4–31.5)	1.00	-	1.00	-
- 1 (*N* = 68)	6.2 (4.9–7.5)	8.56 (5.04–14.53)	< 0.001	10.07 (5.69–17.83)	< 0.001
- 2 (*N* = 64)	2.3 (1.9–2.7)	33.90 (18.76–61.27)	< 0.001	47.56 (24.75–91.38)	< 0.001
First-line therapy					
- Only pembrolizumab (*N* = 124)	6.3 (1.1–8.4)	1.00	-	1.00	-
- Pemetrexed-based (*N* = 75)	8.1 (6.3–9.9)	0.99 (0.71–1.39)	0.981	1.91 (0.22–16.30)	0.553
- Paclitaxel-based (*N* = 17)	8.5 (6.6–10.5)	0.76 (0.38–1.52)	0.448	2.00 (0.21–18.87)	0.543
Corticosteroids^a^					
- No (*N* = 122)	10.0 (7.4–12.7)	1.00	-	1.00	-
- Yes (*N* = 94)	4.1 (2.6–5.6)	1.79 (1.31–2.45)	< 0.001	1.59 (1.08–2.34)	0.018
APAP^b^					
- No (*N* = 154)	8.9 (6.8–10.9)	1.00	-	1.00	-
- Yes (*N* = 62)	3.4 (1.9–5.2)	1.50 (1.07–2.11)	0.016	1.74 (1.17–2.59)	0.006
Systemic antibiotics^c^					
- No (*N* = 176)	8.3 (6.5–10.1)	1.00	-	1.00	-
- Yes (*N* = 40)	3.7 (2.9–4.5)	1.70 (1.16–2.51)	0.007	1.42 (0.88–2.31)	0.148
PPI^d^					
- No (*N* = 158)	7.0 (5.5–8.5)	1.00	-	1.00	-
- Yes (*N* = 58)	7.7 (3.8–11.7)	1.01 (0.70–1.41)	0.998	0.92 (0.68–1.51)	0.921
Benzodiazepines (generic intake)					
- No (*N* = 108)	5.7 (3.4–8.0)	1.00	-	1.00	-
- Yes (*N* = 108)	8.1 (6.4–9.7)	0.92 (0.68–1.26)	0.640	0.73 (0.52–1.01)	0.063
Benzodiazepines (specific intake)					
- No (*N* = 108)	5.7 (3.4–8.0)	1.00	-	1.00	-
- *N*-unsubstituted (*N* = 51)	6.7 (4.1–9.2)	1.31 (0.88–1.94)	0.173	1.21 (0.77–1.89)	0.402
- *N*-substituted (*N* = 57)	9.7 (4.1–15.4)	0.73 (0.50–0.97)	0.041	0.52 (0.34–0.79)	0.002

PFS: progression-free survival; CI: confidence interval; HR: hazard ratio; ECOG PS: Eastern Cooperative Oncology Group Performance Status; PD-L1 TPS: programmed cell death ligand-1 tumor proportion score; BMI: body mass index; RT: radiotherapy; LIPI: lung immune prognostic index; APAP: acetaminophen; PPI: proton pump inhibitors. ^a^ Corticosteroids refer to the use of prednisone or an equivalent drug at a dose of at least 10 mg per day for at least 5 days within the 30 days before the start of treatment, excluding premedication for chemotherapy); ^b^ APAP refers to the use of at least 1,000 mg per day for more than 24 h during the 30 days before the start of treatment; ^c^ systemic antibiotics and ^d^ PPI refer to the use of these medications in the 30 days before the start of treatment

**Table 3:** Analysis of OS

**Covariate**	**Median OS, months (95% CI)**	**Univariate analysis**		**Multivariate analysis**	
		**HR (95% CI)**	***P* value**	**HR (95% CI)**	***P* value**
Age					
- < 70 years (*N* = 105)	13.3 (9.7–16.8)	1.00	-	1.00	-
- ≥ 70 years (*N* = 111)	12.0 (8.7–15.2)	0.86 (0.62–1.18)	0.352	0.83 (0.57–1.23)	0.369
Sex					
- Female (*N* = 74)	11.6 (6.4–16.8)	1.00	-	1.00	-
- Male (*N* = 142)	13.3 (11.1–15.5)	0.82 (0.59–1.13)	0.235	0.79 (0.53–1.18)	0.256
ECOG PS					
- 0–1 (*N* = 175)	13.4 (11.3–15.6)	1.00	-	1.00	-
- 2 (*N* = 41)	7.3 (6.0–8.6)	1.28 (0.84–1.93)	0.239	1.09 (0.67–1.77)	0.727
Histologic subtype					
- Nonsquamous (*N* = 167)	11.1 (7.9–14.2)	1.00	-	1.00	-
- Squamous (*N* = 49)	14.8 (10.2–19.4)	0.69 (0.46–1.04)	0.082	0.65 (0.39–1.09)	0.104
No. of metastatic sites					
- ≤ 2 (*N* = 120)	14.3 (13.1–15.5)	1.00	-	1.00	-
- > 2 (*N* = 96)	9.1 (6.1–12.1)	1.50 (1.09–2.07)	0.013	3.19 (1.76–5.76)	< 0.001
Bone metastasis					
- No (*N* = 172)	13.4 (11.2–15.6)	1.00	-	1.00	-
- Yes (*N* = 44)	9.0 (6.0–11.9)	1.58 (1.08–2.33)	0.018	0.72 (0.43–1.22)	0.234
Brain metastasis					
- No (*N* = 161)	13.3 (11.3–15.4)	1.00	-	1.00	-
- Yes (*N* = 55)	10.8 (4.3–17.3)	0.96 (0.67–1.39)	0.859	0.51 (0.30–0.86)	0.012
Liver metastasis					
- No (*N* = 196)	13.0 (10.7–15.4)	1.00	-	1.00	-
- Yes (*N* = 20)	10.4 (1.9–19.7)	1.25 (0.74–2.11)	0.391	0.51 (0.32–1.19)	0.155
PD-L1 TPS					
- < 1% (*N* = 71)	12.0 (10.2–13.7)	1.00	-	1.00	-
- ≥ 1% and ≤ 49% (*N* = 22)	14.7 (12.3–17.2)	0.92 (0.64–1.33)	0.684	1.57 (0.80–3.11)	0.188
- ≥ 50% (*N* = 123)	9.9 (3.8–16.1)	0.88 (0.50–1.52)	0.651	1.83 (0.21–15.78)	0.582
BMI					
- < 25 kg/m^2^ (*N* = 102)	9.9 (5.8–14.1)	1.00	-	1.00	-
- ≥ 25 kg/m^2^ (*N* = 114)	13.7 (10.1–17.4)	0.63 (0.46–0.87)	0.006	0.69 (0.48–1.01)	0.059
Smoking habits					
- Never (*N* = 20)	4.9 (2.3–7.4)	1.00	-	1.00	-
- Ever (*N* = 196)	13.3 (11.0–15.5)	0.58 (0.35–0.95)	0.033	1.13 (0.62–2.04)	0.679
Previous thoracic RT					
- No (*N* = 188)	11.6 (9.0–14.3)	1.00	-	1.00	-
- Yes (*N* = 28)	16.1 (11.5–20.8)	0.75 (0.47–1.18)	0.214	0.37 (0.22–0.64)	< 0.001
LIPI score					
- 0 (*N* = 84)	28.4 (15.3–41.4)	1.00	-	1.00	-
- 1 (*N* = 68)	11.0 (9.4–12.6)	21.03 (11.25–39.30)	< 0.001	39.00 (17.31–87.87)	< 0.001
- 2 (*N* = 64)	3.3 (2.5–4.2)	> 100 (NA)	< 0.001	> 100 (NA)	< 0.001
First-line therapy					
- Only pembrolizumab (*N* = 124)	9.7 (3.8–15.5)	1.00	-	1.00	-
- Pemetrexed-based (N = 75)	13.3 (10.5–16.0)	0.90 (0.63–1.27)	0.559	0.83 (0.09–7.37)	0.874
- Paclitaxel-based (*N* = 17)	13.3 (11.4–15.1)	0.85 (0.42–1.71)	0.854	1.58 (0.16–15.27)	0.691
Corticosteroids^a^					
- No (*N* = 122)	16.1 (12.4–20.3)	1.00	-	1.00	-
- Yes (*N* = 94)	9.0 (6.5–11.5)	1.76 (1.27–2.43)	0.001	1.79 (1.20–2.66)	0.004
APAP^b^					
- No (*N* = 154)	13.7 (11.2–16.3)	1.00	-	1.00	-
- Yes (*N* = 62)	7.2 (4.2–10.2)	1.41 (1.01–1.99)	0.046	1.37 (0.93–2.04)	0.108
Systemic antibiotics^c^					
- No (*N* = 176)	13.5 (11.2–15.7)	1.00	-	1.00	-
- Yes (*N* = 40)	6.8 (4.7–8.8)	1.63 (1.09–2.43)	0.016	0.69 (0.40–1.18)	0.184
PPI^d^					
- No (*N* = 158)	13.4 (11.1–15.6)	1.00	-	1.00	-
- Yes (*N* = 58)	10.8 (5.6–16.1)	1.01 (0.71–1.43)	0.951	1.09 (0.72–1.63)	0.675
Benzodiazepines (generic intake)					
- No (*N* = 108)	11.7 (8.1–15.2)	1.00	-	1.00	-
- Yes (*N* = 108)	13.7 (10.6–16.9)	0.91 (0.66–1.25)	0.566	0.69 (0.48–1.01)	0.058
Benzodiazepines (specific intake)					
- No (*N* = 108)	11.7 (8.1–15.2)	1.00	-	1.00	-
- *N*-unsubstituted (*N* = 51)	9.1 (6.0–12.2)	1.57 (1.05–2.34)	0.025	1.92 (1.20–3.06)	0.006
- *N*-substituted (*N* = 57)	16.2 (10.4–21.9)	0.64 (0.43–0.94)	0.026	0.58 (0.38–0.88)	< 0.001

OS: overall survival; CI: confidence interval; HR: hazard ratio; ECOG PS: Eastern Cooperative Oncology Group Performance Status; PD-L1 TPS: programmed cell death ligand-1 tumor proportion score; BMI: body mass index; RT: radiotherapy; LIPI: lung immune prognostic index; APAP: acetaminophen; PPI: proton pump inhibitors. ^a^ Corticosteroids refer to the use of prednisone or an equivalent drug at a dose of at least 10 mg per day for at least 5 days within the 30 days before the start of treatment, excluding premedication for chemotherapy); ^b^ APAP refers to the use of at least 1,000 mg per day for more than 24 h during the 30 days before the start of treatment; ^c^ systemic antibiotics and ^d^ PPI refer to the use of these medications in the 30 days before the start of treatment

## Discussion

The current study investigated the potential impact of BZD use on the survival outcomes of patients with metastatic NSCLC undergoing treatment with pembrolizumab. The results indicated that generic BZD exposure in the time frame immediately preceding and following the onset of immune checkpoint blockade did not influence PFS or OS. However, we observed that patients prescribed *N*-substituted BZDs had a significant reduction in the risk of disease progression and mortality compared to those not using BZDs or using *N*-unsubstituted compounds. In addition, the latter patients experienced the worst survival outcome. Univariate analysis also showed that the survival benefit was limited only to patients with high levels of PD-L1 expression who had received pembrolizumab alone. These novel findings prompt further discussion.

The study utilized a research methodology involving real-world data from a single medical center. This framework allowed a thorough review of healthcare records, reflecting a prescribing attitude that may not be extended elsewhere. However, such an approach has been recognized for its value in oncology research for addressing issues that may be challenging to investigate prospectively [27]. Through a retrospective analysis, the study sought to examine for the first time the impact of BZD exposure on outcomes of immune checkpoint blockade. To ensure an optimal prognostic balance in the dataset, we performed PSM considering all relevant clinical, pathological, and pharmacological factors in accordance with established research protocols [28]. Furthermore, the close adherence of the data to the National Drug Agency registry enhances the reliability of our findings [19].

In recent years, the role of concomitant medications has been extensively investigated in patients with advanced cancer receiving immune checkpoint inhibitors. Exposure to concurrent drugs is an essential component of the exposome, as they can influence the anticancer immune response through several mechanisms, including direct effects on immune cells, modulation of TME, and modification of the microbiome [29]. Although substantial preclinical evidence has shown that BZDs can interact at different levels, most studies have not considered these drugs as relevant external factors for the host during immune checkpoint blockade. Even the most recent meta-analyses, which applied a more comprehensive inquiry methodology relying on the umbrella review, did not refer to the concomitant use of BZDs [30, 31]. Currently, only two studies are available to compare with our findings. The first retrospectively evaluated the effect of beta blockers on clinical outcomes in patients with advanced NSCLC who were treated with immune checkpoint inhibitors. Patients were also assessed for concurrent exposure to other medications, including BZDs in 36% of cases. Consistent with our results, the authors reported that generic BZD intake was associated with not significant trends toward worse PFS and OS [32]. Recently Montégut et al. [33] provided valuable insights in this regard. The study showed that the acyl-CoA-binding protein (ACBP) acts as a positive allosteric modulator on the GABA_A_ receptor through a specific binding site that is shared by diazepam and other BZDs. The authors found that ACBP exerts immunosuppressive properties, and its antibody-mediated neutralization has immunostimulatory effects, improving the efficacy of immunotherapy and chemoimmunotherapy in mouse models. They also demonstrated that administration of diazepam in mice abrogates the favorable effects of anti-ACBP antibodies on cancer chemoimmunotherapy, supporting the suggestion that diazepam may act as an exogenous immunosuppressant. Montégut et al. [33] also showed, in a small cohort of advanced NSCLC patients undergoing immunotherapy or chemoimmunotherapy, that concomitant BZD intake was associated with significantly reduced PFS and a trend toward shorter OS. These findings appear partially consistent with our subgroup analysis showing that it is not so much the drug class affecting survival but rather the molecular structure of BZDs that modulates the efficacy of immune checkpoint inhibitors with opposite effects. A comprehensive study by Cornwell et al. [18] provided us with additional interpretive insights. This research focused on exploring the relationship between BZDs and survival outcomes of cancer patients. Among patients with pancreatic cancer who were undergoing chemotherapy, the use of lorazepam was linked to shortened PFS, whereas the intake of alprazolam was associated with an improvement in the same outcome. In comparison to both alprazolam and patients who did not take BZDs, lorazepam exposure resulted in poorer survival outcomes across many other cancer types. The authors provided additional data obtained from in vivo mouse models. Accordingly, the harmful impact of lorazepam relied on positive allosteric modulation of ovarian cancer G-protein-coupled receptor 1 (OGR1 or GPR68). The downstream effects of enhanced GPR68 signaling would have occurred only after exposure to lorazepam and other *N*-unsubstituted BZDs, promoting the transition of TME into an immunosuppressive phenotype through increased IL-6 expression. Conversely, alprazolam and other *N*-substituted BZDs were found to decrease IL-6 expression in a GPR68-independent manner. The expression of GPR68 is highly upregulated in several cancer types, including different histological subtypes of NSCLC [34]. GPR68 regulates signal transduction pathways that are essential for several processes in tumor biology, such as cell proliferation, inhibition of apoptosis, invasion, angiogenesis, and metastasis [35, 36]. Emerging evidence has revealed that GPR68 is also overexpressed in T cells and can modulate tumor immune evasion [37]. These preclinical data indicate that GPR68 expression in CD8^+^ T lymphocytes plays a dominant role in suppressing antitumor immunity [38, 39]. Although clinical evidence that GPR68-mediated signaling may have a predictive role in cancer immunotherapy is lacking, the latter considerations may provide an interpretive key to our results. We could argue that exposure to *N*-unsubstituted BZDs blunts the efficacy of immune checkpoint inhibition therapy through selective positive allosteric modulation of GPR68 and a subsequent increase in IL-6 expression [40, 41]. Conversely, exposure to *N*-substituted BZDs, which lack affinity for GPR68, would even result in decreased IL-6 levels and improved outcomes of immune checkpoint blockade [42]. These putative interactions could also explain the absence of significant differences in survival among patients with lower PD-L1 TPS who received the combination of pembrolizumab and cytotoxic chemotherapy. Since platinum analogs [43, 44], pemetrexed [45], and paclitaxel [46] can directly impact on the levels of IL-6 expression, it is conceivable that the effects of cytotoxic chemotherapy were predominant, abrogating the modulating potential of different BZD compounds in this specific subgroup of patients.

Our study has various constraints that must be acknowledged. Primarily, the research methodology was retrospective in nature and carried out at a singular institution. Despite utilizing an established prospective registry as the primary data source, employing predefined assessment schedules, and adhering to uniform treatment decision criteria, we cannot disregard the possibility of selection bias. Efforts have been made to mitigate this bias by enrolling consecutive patients and applying thorough criteria for PSM, but the presence of confounding variables may have influenced the results to some extent. Second, radiological assessment of PFS was blinded but not performed by an independent panel, implying an overestimate of this outcome. Third, retrospective data on concomitant medications were derived from internal records on medical and pharmaceutical prescriptions. While this approach is affordable, it may lack the accuracy associated with data obtained directly from a clinical trial. Finally, we cannot neglect the risk of alpha inflation arising from univariate multiple comparisons. Although our multivariate analysis was comprehensive, including all potential prognostic factors to mitigate the risk of false-negative results in a new experimental setting, the possibility of false-positive results remains inherent to this methodology. Considering these limitations, it is important to note that our findings should not be considered conclusive or generalizable.

BZDs are structurally complex molecules with pharmacodynamic properties that are not fully known. The current research indicates that concomitant exposure to these medications could affect the efficacy of immune checkpoint blockade in advanced NSCLC. Regarding patients with a high PD-L1 TPS who are exclusively given pembrolizumab, BZDs appear to influence survival outcomes in opposite directions depending on their specific composition. Preclinical evidence suggests that the varying ways in which BZDs modulate IL-6 expression levels within the TME could underlie the diverse effects on antitumor immune responses. The relevant signaling pathway would not involve stimulation of on-target BZD receptors, including GABA_A_ or TSPO, but rather allosteric modulation of an off-target binding site, such as GPR68. From a clinical standpoint, BZDs remain the most easily and frequently prescribed psychotropic drugs in advanced cancer patients with different indications. Our results may not influence prescribing attitudes regarding dosage or duration of treatment, but they imply that treating physicians should not underestimate specific exposure to BZDs at the onset of immune checkpoint blockade. In this regard, the choice or modification of concomitant therapy with BZD should rely on specific molecular characteristics of these medications. The limitations of this study are crucial to recognize, and there is insufficient evidence to make a thorough comparison. This implies the need for further verification of our findings in the separate series.

## Abbreviations

ACBP: acyl-CoA-binding protein
APAP: acetaminophen
BMI: body mass index
BZD: benzodiazepine
CI: confidence interval
ECOG PS: Eastern Cooperative Oncology Group Performance Status
GABA: γ-aminobutyric acid
HR: hazard ratio
LIPI: lung prognostic immune index
NSCLC: non-small-cell lung cancer
OS: overall survival
PD-L1: programmed cell death ligand-1
PFS: progression-free survival
PPI: proton pump inhibitors
PSM: propensity score matching
SD: standard deviation
SMD: standardized mean difference
TME: tumor microenvironment
TPS: tumor proportion score

## Supplementary materials

The supplementary material for this article is available at: https://www.explorationpub.com/uploads/Article/file/1002287_sup_1.pdf.

## Declarations

### Availability of data and materials

The raw data supporting the conclusions of this manuscript will be made available by the authors to any qualified researcher on reasonable request.

### Copyright

© The Author(s) 2025.
