# Supplementary material for: Concomitant exposure to benzodiazepines during pembrolizumab-based therapy for advanced non-small-cell lung cancer: a propensity-score matched analysis of monitoring agency data
Source: Explor Target Antitumor Ther. 2025 Jan 20;6:1002287. doi: 10.37349/etat.2025.1002287 (PMC11886376; doi:10.37349/etat.2025.1002287)
Supplement: Supplementary file 1 [file 1002287_sup_1.pdf]

Supplementary Material

Concomitant exposure to benzodiazepines during pembrolizumab-based therapy for advanced non-small-cell lung cancer: a propensity-score matched analysis of monitoring agency data

Fabrizio Nelli[1*], Enzo Maria Ruggeri[2], Antonella Virtuoso[1], Diana Giannarelli[3], Armando Raso[4], Federica Natoni[5], Gloria Pessina[5], Daniele Remotti[6], Mario Giovanni Chilelli[2], Carlo Signorelli[2], Agnese Fabbri[2]

[1]Department of Oncology and Hematology, Thoracic Oncology Unit, Central Hospital of Belcolle, 01100 Viterbo, Italy

[2]Department of Oncology and Hematology, Medical Oncology Unit, Central Hospital of Belcolle, 01100 Viterbo, Italy

[3]Biostatistics Unit, Scientific Directorate, Fondazione Policlinico Universitario A. Gemelli, IRCCS, 00136 Rome, Italy

[4]Department of Oncology and Hematology, Thoracic and Interventional Radiology, Central Hospital of Belcolle, 01100 Viterbo, Italy

[5]Department of Oncology and Hematology, Molecular Biology and Genetics, Central Hospital of Belcolle, 0100 Viterbo, Italy

[6]Department of Oncology and Hematology, Pathology Unit, Central Hospital of Belcolle, 01100 Viterbo, Italy

Table S1. Patient characteristics depending on benzodiazepine classification (N=108)

Variable	N-substituted (N=57)	N-unsubstituted (N=51)	P value
Drug			-
- alprazolam	28 (49.1%)	-	
- diazepam	17 (29.8%)	-	
- bromazepam	8 (14.0%)	-	
- triazolam	4 (7.0%)	-	
- lorazepam	-	23 (45.1%)	
- clonazepam	-	11 (21.6%)	
- delorazepam	-	10 (19.6%)	
- lormetazepam	-	5 (9.8%)	
Median length of therapy before ICI initiation, days (IQR)	44 (37-71)	51 (39-67)	0.348
Therapeutic indication			0.979
- Generalized anxiety disorder	26 (45.6%)	22 (43.1%)	
- Anxiety-depressive disorder	14 (24.6%)	13 (25.5%)	
- Insomnia	11 (19.3%)	9 (17.6%)	
- Panic disorder	2 (3.5%)	3 (5.9%)	
- Others	4 (7.0%)	4 (7.8%)	
Age			
- Mean (SD), years	71 (10.7)	69 (6.7)	0.266
- ≥70 years	32	22	0.177
Sex			0.235
- Female	23 (40.3%)	15 (29.4%)	
- Male	34 (59.7%)	36 (70.6%)	
ECOG PS			0.783
- 0-1	47 (82.5%)	41 (80.4%)	
- 2	10 (17.5%)	10 (19.63%)	
Histologic subtype			0.757
- Nonsquamous	45 (78.9%)	39 (76.5%)	
- Squamous	12 (21.1%)	12 (23.5%)	
No. of metastatic sites			0.023
- ≤2	37 (64.9%)	22 (43.1%)	
- >2	20 (35.1%)	29 (56.9%)	
Bone metastases	11 (19.3%)	12 (23.5%)	0.592
Brain metastases	17 (29.8%)	11 (21.6%)	0.328
Liver metastases	4 (7.0%)	5 (9.8%)	0.601
PD-L1 TPS			0.104
-<1%	16 (28.1%)	19 (37.3%)	
- ≥1% and ≤49%	11 (19.3%)	3 (5.9%)	
- ≥50%	30 (52.6%)	29 (56.9%)	
BMI			
- Mean (SD), (kg/m^2)	25.1 (4.5)	26.1 (4.5)	0.958
- ≥25	29 (50.9%)	29 (54.9%)	0.676
Smoking habits			0.383
- never	6 (10.5%)	3 (5.9%)	
- ever	51 (89.5%)	48 (94.1%)	
Previous thoracic RT	16 (28.1%)	3 (5.9%)	0.003
Autoimmune disease	4 (7.0%)	-	0.054

LIPI score<br>- 0<br>- 1<br>- 2	 22 (38.6%)<br>18 (31.6%)<br>17 (29.8%)	 18 (35.3%)<br>18 (35.3%)<br>15 (29.4%)	0.908
First-line therapy<br>- Only pembrolizumab<br>- Pemetrexed-based<br>- Paclitaxel-based	 30 (52.6%)<br>22 (38.6%)<br>5 (8.8%)	 30 (58.8%)<br>17 (33.3%)<br>4 (7.8%)	0.811
Corticosteroids [a]	21 (36.8%)	28 (54.9%)	0.060
APAP [b]	18 (31.6%)	16 (31.4%)	0.982
Systemic antibiotics [c]	9 (15.8%)	11 (21.6%)	0.440
PPI [d]	17 (29.8%)	12 (23.5%)	0.461

SD, standard deviation; ECOG PS, Eastern Cooperative Oncology Group Performance Status; PD-L1 TPS, programmed cell death ligand-1 tumor proportion score; BMI, body mass index; RT, radiotherapy; LIPI, lung immune prognostic index; APAP, acetaminophen; PPI, proton pump inhibitors

[a] Corticosteroids indicate intake of prednisone equivalent ≥10 mg daily for at least 5 days within the 30 days prior to the start of treatment (excluding premedication for chemotherapy); [b] APAP indicates a therapeutic intake of at least 1000 mg per day for more than 24 hours during the 30 days prior to the start of treatment; [c] systemic antibiotics indicate a therapeutic intake in the 30 days prior to the start of treatment; [d] PPI indicates any intake at the start of treatment.

Table S2. Logistic regression analysis for factors associated with specific benzodiazepine intake (N-unsubstituted vs. N-substituted compounds, N=108)

Covariate	Univariate analysis		Multivariate analysis	
	OR (95% CI)	P value	OR (95% CI)	P value
Age<br>- ≥70 years (vs <70 years)	0.59 (0.27-1.27)	0.179	0.29 (0.09-0.88)	0.029
Sex<br>- Female (vs. male)	1.62 (0.72-3.62)	0.236	1.74 (0.58-4.93)	0.325
ECOG PS<br>- 2 (vs. 0-1)	1.14 (0.43-3.02)	0.783	0.72 (0.18-2.79)	0.638
Histologic subtype<br>- Squamous (vs. non-squamous)	1.15 (0.46-2.86)	0.757	1.22 (0.30-4.97)	0.774
No. of metastatic sites<br>- >2 (vs. ≤2)	2.43 (1.12-5.30)	0.024	29.10 (3.80->100)	0.001
Bone metastases<br>- yes (vs. no)	1.28 (0.51-3.23)	0.592	0.32 (0.07-1.49)	0.142
Brain metastases<br>- yes (vs. no)	0.64 (0.26-1.55)	0.330	0.10 (0.01-0.68)	0.018
Liver metastases<br>- yes (vs. no)	1.44 (0.36-5.68)	0.602	0.23 (0.01-3.24)	0.277
PD-L1 TPS<br>-<50% (vs ≥50%)	1.28 (0.60-2.75)	0.518	0.17 (0.02-1.22)	0.079
BMI (kg/m^2)<br>- ≥25 (vs. <25)	1.17 (0.55-2.50)	0.676	1.19 (0.42-3.38)	0.739
Smoking habits<br>- ever (vs. never)	1.88 (0.44-7.95)	0.390	3.13 (0.41-23.52)	0.267
Previous thoracic RT<br>- yes (vs. no)	0.16 (0.04-0.58)	0.006	0.08 (0.01-0.49)	0.006
LIPI score				
- 0	1.00 (reference)	-	1.00 (reference)	0.905
- 1	1.22 (0.49-3.01)	0.663	0.97 (0.30-3.06)	0.964
- 2	1.07 (0.42-2.74)	0.874	0.76 (0.21-2.66)	0.668
Corticosteroids [a]<br>- yes (vs. no)	2.08 (0.96-4.51)	0.061	1.64 (0.55-4.89)	0.373
APAP [b]<br>- yes (vs. no)	0.99 (0.43-2.23)	0.982	0.93 (0.29-2.93)	0.904
Systemic antibiotics [c]<br>- yes (vs. no)	1.46 (0.55-3.89)	0.442	1.27 (0.29-5.43)	0.747
PPI [d]<br>- yes (vs. no)	0.72 (0.30-1.71)	0.462	0.30 (0.08-1.09)	0.069

OR, odds ratio; CI, confidence interval; ECOG PS, Eastern Cooperative Oncology Group Performance Status; PD-L1 TPS, programmed cell death ligand-1 tumor proportion score; BMI, body mass index; RT, radiotherapy; LIPI, lung immune prognostic index; APAP, acetaminophen; PPI, proton pump inhibitors

[a] Corticosteroids indicate intake of prednisone equivalent ≥10 mg daily for at least 5 days within the 30 days prior to the start of treatment (excluding premedication for chemotherapy); [b] APAP indicates a therapeutic intake of at least 1000 mg per day for more than 24 hours during the 30 days

prior to the start of treatment; [c] systemic antibiotics indicate a therapeutic intake in the 30 days prior to the start of treatment; [d] PPI indicates any intake at the start of treatment.
